# Significant Decrease in Heavy Metals in Surface Sediment after Ten-Year Sustainable Development in Huaxi Reservoir Located in Guiyang, Southwestern China

**DOI:** 10.3390/ijerph18147684

**Published:** 2021-07-20

**Authors:** Xiangyang Zhou, Kejia Zhou, Rong Liu, Shanggui Sun, Xinqiang Guo, Yanni Yang, Lixia Chen, Kun Zou, Wenjuan Lei

**Affiliations:** 1 College of Resources and Environmental Engineering, Guizhou University, Guiyang 550025, China; xyzhou6@gzu.edu.cn (X.Z.); jiazhou9711@163.com (K.Z.); liur960804@163.com (R.L.); Sun18285090582@163.com (S.S.); xqg850402@126.com (X.G.); yangyn0417@163.com (Y.Y.); lxchen@gzu.edu.cn (L.C.); zoukkun@126.com (K.Z.); 2 Key Laboratory of Karst Geological Resources and Environment (Ministry of Education), Guizhou University, Guiyang 550025, China; 3 College of Tea Science, Guizhou University, Guiyang 550025, China

**Keywords:** heavy metals, sediment, karst area, Huaxi reservoir

## Abstract

In the Karst area of southwestern China, the heavy metals in the sediment of a reservoir are determined by both human activities and the high background values. Thus, this study explores the change of heavy metals in surface sediment after ten-year sustainable development in the upstream areas of a reservoir, Huaxi Reservoir, located in Guiyang of southwestern China, then evaluates the risk of these heavy metals to water environment systematically and finally identifies the sources in both 2019 and 2009. The results reveal that all of the measured heavy metals decrease dramatically and their spatial distributions change from the increase-decrease pattern to decrease-increase pattern, implying different locations of main source input. The risk indices based on the total or average content and relative or reference values have decreased to the lowest level. However, those indices calculated from the absolute content of each metalloid still show a low or a moderate risk because of the high background value, such as As and Cr. Moreover, although only one main source of heavy metals is identified in both 2019 and 2009, the risk from human activities still cannot be neglected because agricultural production and infrastructure construction would promote the weathering of soil and then these heavy metals from the soil will be brought into the reservoir with the rainfall-runoff process. The high background value of specific heavy metals, e.g., As and Cr would still exert some challenges to the water environment protections because the non-point source input of heavy metal cannot be controlled easily by promulgating a series of bans. These results provide important reference for creating the policies of water environment protection, especially in some Karst area of southwestern China that exhibits high background value of heavy metals.

## 1. Introduction

Heavy metals are one of the main sources of pollution to water body. Generally, heavy metals originate from natural sources and anthropogenic activities [1]. Mining, smelting, traffic, machinery manufacturing, sewage, chemical fertilizer and pesticide are the main contributors to environment pollution by heavy metal [2,3,4,5]. Once these heavy metals are discharged into water environment, they could threaten the ecosystem and public health due to the bioaccumulation or biomagnification in the aquatic food web [6,7,8,9,10,11]. Particularly, the risk level could be higher in areas with high background values such as Karst region of Guizhou Province in southwestern China [12,13,14,15,16]. 

Sediments, which are one of the key components of aquatic ecosystem, not only provide habitats and foods for benthic organisms but have also been considered as sinks and secondary sources of heavy metals in the water environment [17,18,19]. The reason is that more than 90% of total heavy metals load in the aquatic environment is bound to suspend matter and then settle down to the bottom of the sediment [5,11,20]. However, the contaminated sediment would release heavy metals into the overlying water when the bottom environment is disturbed [11] and then pollute the water and pose environmental risk to the aquatic ecosystems [21]. A large number of research have reported that the heavy metal contaminated sediment has become a serious threat to aquatic ecosystems, such as river [9,19,22], lake [11,23], estuarine [24], wetland [25] and reservoirs [26]. Thus, sediments have been widely used as aquatic environmental indicators of the present contamination characteristics of trace metals [27]. 

In order to quantify the environmental risk of the heavy metals in sediment, several empirical and statistical indices have also been applied to evaluate the pollution status and ecological risk. For example, geo-accumulation index (*I_geo_*), enrichment factor (*EF*), potential ecological risk index (RI), contamination factor (*CF*) and sediment quality guidelines (SQG) have been employed to assess the heavy metal contamination [21,28,29]. Hazard index (*HI*) and carcinogenic risk or the lifetime cancer risk (*LCR*) have also been adopted to assess potential human health risks [30]. Moreover, the Pearson’s correlation coefficient (PCC), principal component analysis (PCA) and hierarchical cluster analysis (*HCA*) are the main statistical methods for recognizing the origin and the evaluation of the load contaminated status of heavy metals [21,22,23]. 

Reservoirs play an important role in the functions of irrigation, drinking water, control flooding and hydropower [31]. In order to protect the water environment, many restoration projects have been executed for several decades in China, such as shutting down polluting enterprises, rationally use fertilizers and pesticides, strictly regulating emission standards and turning agricultural land back into forests. After taking a series of tools, the content of some heavy metals decreases dramatically. However, whether there are still environmental risks that still exists are not clear. 

Therefore, the objectives of this study are to explore the change of heavy metals in sediments after ten years of sustainable development in the upstream areas of reservoir, Huaxi Reservoir, located in Guiyang of southwestern China with high background values of specific heavy metals such as As and Cr and then to evaluate the risk of these heavy metals to the water environment. The results would provide important reference for making policies of water environment protection, especially in the areas where heavy metals exhibit high background values. 

## 2. Materials and Methods

### 2.1. Study Area 

**Figure 1:**
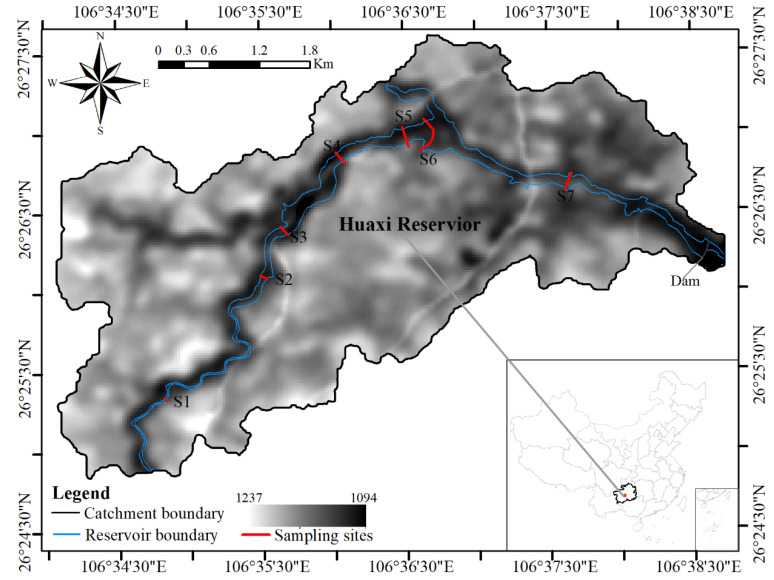
Sampling sites and DEM of in the catchment of Huaxi Reservoir.

The study area, Huaxi Reservoir, is located in the Guiyang of southwestern China (N26°25″ N; 106°40″ E), as shown in Figure 1. This area features subtropical humid climate with the average temperature of 15.3 °C and annual precipitation of 1129.5 mm. The reservoir was built in 1958. The storage capacity reached 31.4 million m^3^ after the latest upgrade in 2008. The maximum height of the dam is 51.6 m and the length of the dam is 384 m. The design flood level is 1143.32 m. The main functions of the reservoir are controlling floods and water supply because the main areas of the Guiyang City and Huaxi District are in the downstream area of the dam. The ability of controlling floods can prevent the extreme risk level of once-in-a-century. There is little agricultural purpose with the process of urbanization. Recreational activities were banned in the reservoir in 2013. In the upstream of the dam, the catchment covers an area of 176 km^2^ and features the city mixed with a series of hills. The average height is about 1165 m with the range of 1094 m to 1237 m based on DEM. As shown in Figure 1, the length of river is about 14.42 km in the upstream areas. 

With urbanization in the last ten years, the area of urban land increased from 7.73% to 16.86%, while the others show a more or less decreasing trend. Based on the investigations in 2009, the main sources of pollution were domestic sewage and agricultural non-point source pollution in the whole catchment and infrastructure constructions in the middle stream areas. At present, most of the domestic sewage is collected systematically, farming activities have decreased substantially because of the development of tourism and there are still some infrastructure constructions in the upstream areas while most of the infrastructure constructions in the middle stream have been completed.

### 2.2. Sample Collection 

In this study, surface sediments (0–5 cm) in Huaxi Reservoir are sampled from 7 typical transects in April 2019. These samples are collected by a stainless-steel hand shovel covered by rubber from upstream to downstream and the location of sampling transects are also shown in Figure 1. In order to evaluate the change of heavy metals in sediment and the restoration of aquatic environment of Huaxi Reservoir, the sampling profiles are kept the same as or close to the previous latest study [32]. 

### 2.3. Sample Analysis

The method of sample analysis is based on the standard issued by Ministry of Environment Protection of the People’s Republic of China (HJ832-2017) [33]. The sediment samples are naturally air-dried at room temperature and then pulverized in an agate mortar and filtered through a sieve of 100 mesh nylon sieve. For heavy metals, approximately 0.10 g of dry sediment sample is digested with acid (6 mL HNO_3_, 2 mL HCl and 2 mL HF) and is incubated in the microwave (Mars-40TFM) of 200 °C for 30 min. After digestion, the resulting solutions are diluted to 50 mL ultrapure water and the analysis was performed by ICP-MS (Thermo Fisher X2, Waltham, MA, USA). Three duplicates are measured in the experimental analysis.

### 2.4. Methods for Evaluation of Pollution Risk

#### 2.4.1. Sediment Quality Guidelines

Sediment quality guidelines (SQGs) are widely used to assess the sediment toxicity [18,22,34]. According to the recommendation of MacDonald et al. [35], the threshold effect level (*TEL*) is used to identify the contaminant concentration below which there is no adverse effects on sediment-dwelling organisms. The probable effect level (*PEL*) and the severe effect level (*SEL*) are used to identify the concentration above which there are possible adverse effects on sediment-dwelling organisms [22,25]. The threshold values of these indices are presented in Table 1.

#### 2.4.2. Geo-Accumulation Index (*I_geo_*)

Geo-accumulation index, *I_geo_*, is widely used to evaluate contamination by correlating the measured current concentration of metals with their background concentrations. The calculation of geo-accumulation index (*I_geo_*) is as follows:

(1) $$I_{geo}=log_{2}\frac{C_{n}}{1.5\;B_{n}}$$

where *C_n_* is the current measured concentration of heavy metals in sediment and *B_n_* is the geochemical background concentration [41]. This study refers the soil metal concentration of Guizhou Province as the sediment background [40], i.e., 95.9 mg/kg for Cr, 20 mg/kg for As, 0.66 mg/kg for Cd, 32 mg/kg for Cu, 99.5 mg/kg for Zn and 35.2 mg/kg for Pb. The constant 1.5 is a matrix correction for lithogenic effects [2]. The *I*_geo_ index is divided into seven classes: *I_geo_* ≤ 0, unpolluted; 0 < *I_geo_* < 1, unpolluted to moderately polluted; 1 < *I_geo_* < 2, moderately polluted; 2 < *I_geo_* < 3, moderately to strongly polluted; 3 < *I_geo_* < 4, strongly polluted; 4 < *I_geo_* < 5, strongly to very strongly polluted; and 5 ≤ *I_geo_*, very strongly polluted.

#### 2.4.3. Contamination Factor (*CF*) 

Contamination factor (*CF*) is usually used to disclose the contamination level of potential toxic elements in sediments and the calculation of *CF* is described as follows [42,43]:

(2) CF=CmetalCbackground

where *C_metal_* is the average concentration of heavy metals in the sediments and *C_background_* is geochemical background concentration. The *CF* index can be divided into four classes: low degree with *CF* < 1; moderate contamination with 1 < *CF* < 3; considerable contamination with 3 ≤ *CF* < 6; and very high contamination with *CF* > 6.

#### 2.4.4. Potential Ecological Risk Index ($E_{r}^{i}$)

Potential ecological risk index ($E_{r}^{i}$) aims to evaluate the degree of pollution in sediment and to determine the pollution [42]. The calculation of $E_{r}^{i}$ is given below:

(3) $$E_{r}^{i}=T_{r}^{i}\times \left(C_{n}/C_{ref}\right)$$

where *C_n_* is the measured concentration of metal, *C_ref_* is the reference value of metal, $T_{r}^{i}$ is the toxic response factor, i.e., 30 for Cd, 10 for As, 5 for Cu, Pb and Ni, 2 for Cr and 1 for Zn. According to the study of Maanan et al. [44], the risk range and their corresponding classifications are as follows: low ecological risk with $E_{r}^{i}$ < 40; medium ecological risk with 40 ≤ $E_{r}^{i}$ < 160; high ecological risk with 160 ≤ $E_{r}^{i}$ < 320; and significantly high ecological risk with 320 ≥ $E_{r}^{i}$.

#### 2.4.5. Enrichment Factor (*EF*)

Enrichment factor (*EF*) is an effective tool to evaluate the pollution degree of heavy metals driven by anthropogenic or geologic forces in sediments [45,46,47]. It is a normalization method suggested by Sinex and Helz [48] and is used in standardizations of the acquired heavy metal content in sediments with respect to a reference metal that is either *Fe* or Al [49]. In this study, *Fe* is used as a reference metal for geochemical normalization. The *EF* is calculated by the following formula:

(4) $$EF=\frac{C_{n}/Fe_{n}}{C_{background}/Fe_{background}}$$

where *C_n_* is the measured concentration of heavy metal, *C_background_* is its corresponding background value and *Fe_n_* and *Fe_background_* are the concentrations of *Fe* in the sample and its corresponding background value, respectively. The sediments could be divided into six classes based on the *EF* value: *EF <* 1.5, no enrichment; 1.5 < *EF* < 2, slight enrichment; 2 < *EF* < 5, moderate enrichment; 5 < *EF* < 20, severe enrichment; 20 < *EF* < 40, highly severe enrichment; and 40 < *EF*, extremely severe enrichment [50].

#### 2.4.6. Toxic Risk Index (*TRI*)

Toxic risk index (*TRI*) method depends on the *TEL* and *PEL* effects for the toxic risk evaluation of heavy metals in sediments [22]. The *TRI* calculation formula is described as the following.

(5) TRIi=(CiTEL)2+(CiPEL)22

The following equation is used to calculate the integrated toxic risks of heavy metals in sediments:

(6) $$TRI=\overset{n}{\underset{i=1}{\sum }}TRI_{i}$$

where $TRI_{i}$ is the toxic risk index of a single heavy metal, *C_i_* is current heavy metal concentration in the sediment sample, *n* is the number of metals and *TRI* is the integrated toxic risk index. The classification of *TRI* is the following: *TRI* ≤ 5, no toxic risk; 5< *TRI* ≤10, low toxic risk; 10 < *TRI* ≤ 15, moderate toxic risk; 15 < *TRI* ≤ 20, considerable toxic risk; and *TRI* > 20, very high toxic risk [22,51]. 

#### 2.4.7. Modified Hazard Quotient (*mHQ*)

Metal concentration in sediments and the synoptic adverse ecological effect distributions for the threshold levels (*TEL*, *PEL* and *SEL*) were reported by MacDonald et al. [35]. The method of modified hazard quotient (*mHQ*) is used to assess the degree of risk posed by heavy metals to the aquatic environment and the sediment dwelling organisms. The following equation is used to calculate the value of *mHQ* [22].

(7) $$mHQ=\lfloor C_{i}\left(\frac{1}{TEL_{i}}+\frac{1}{PEL_{i}}+\frac{1}{SEL_{i}}\right)\rfloor^{\frac{1}{2}}$$

The *mHQ* rank comprises of eight classes: *mHQ* < 0.5, nil to very low severity of contamination; 0.5 < *mHQ* < 1.0, very low severity of contamination; 1.0 < *mHQ* < 1.5, low severity of contamination; 1.5 < *mHQ* < 2.0, moderate severity of contamination; 2.0 < *mHQ* < 2.5, considerable severity of contamination; 2.5 < *mHQ* <3.0, high severity of contamination; 3.0 < *mHQ* < 3.5, very high severity of contamination; *mHQ* > 3.5, extreme severity of contamination.

#### 2.4.8. Ecological Contamination Index (*ECI*)

The *ECI* is an aggregative empirical approach that estimates the risks associated with an ecosystem using a source specific factor derived primarily from principal component analysis (PCA)/factor analysis (FA). The *ECI* is calculated in the following [18]:

(8) $$ECI=B_{n}\sum_{i=1}^{n}mHQ_{i}$$

where *B_n_* is the reciprocal of derived eigenvalue of heavy metal concentrations. The proposed ranking of risks posed by heavy metals to ecological systems using the *ECI* is the following: *ECI* > 7, extremely contaminated; 6 < *ECI* < 7, highly contaminated; 5 < *ECI* < 6, considerably to highly contaminated; 4 < *ECI* < 5, moderately to considerably contaminated; 3 < *ECI* < 4, slightly to moderately contaminated; 2 < *ECI* < 3, uncontaminated to slightly contaminated; *ECI* < 2, uncontaminated [18].

#### 2.4.9. Potential Human Health Risk Assessment

Health risk assessment is a commonly used method to estimate the risk posed to humans due to exposure to certain contaminants of known amounts [30,52]. Heavy metal exposure to humans can occur through three major pathways either by means of (1) direct oral ingestion of heavy metal particles, (2) inhalation of the heavy metal particles through the mouth and nose and (3) dermal absorption of the particles attached to exposed skin [30,53].

The chronic daily intake (*CDI*) (mg/kg/day) of contaminant was applied to estimate the health risks via ingestion, inhalation and dermal contact path ways on both adults and children. The equations estimating the *CDI* are described as follows [30]:

(9) $$CDI_{ingest}=\frac{C_{sed}\times IngR\times EF\times ED\times CF}{BW\times AT}$$

(10) $$CDI_{inhale}=\frac{C_{sed}\times InhR\times EF\times ED}{BW\times AT\times PEF}$$

(11) $$CDI_{dermal}=\frac{C_{sed}\times SA\times EF\times ED\times CF\times AF_{sed}\times ABS}{BW\times AT}$$

where *C_sed_* is the concentration of heavy metal in sediment (mg/kg), *IngR* indicates the ingestion rate of the soil (100 mg/day for adult and 200 mg/day for children), *EF* is the exposure frequency (350 days/year), *ED* is the exposure duration (24 years for adult and 6 years for children), *BW* is the average body weight (70 kg for adult and 15 kg for children), *AT* is the averaging time (365 × *ED*), *CF*, as shown in 2.4.3, is the conversion factor (1 × 10^−6^ kg/mg), *InhR* is the inhalation rate (20 mg/cm^2^), *PEF* is the particle emission factor (1.36 × 10^9^ m^3^/kg), *SA* is the surface area of the skin that is in contact with the soil (5700 cm^2^/event), *AF_sed_* is the skin adherence factor (0.07 mg/cm^2^) and *ABS* is the dermal absorption factor (0.001). The exposure factors referenced by Yuswir et al. [54] are based on the values documented in USEPA [55].

The hazard index (*HI*) was used to evaluate the cumulative non-carcinogenic risk. *HI* equals to the sum of hazard quotient (*HQ*), as shown in Equations (12) and (13):

(12) $$HQ=\frac{CDI}{RfD}$$

(13) $$HI=\sum HQ=HQ_{ing}+HQ_{inh}+HQ_{dermal}$$

where *RfD* refers to the reference dose based on USEPA [56]. The values of *R*_f_*D* are different from one another, i.e., 0.0371 for Cu, 0.0035 for Pb, 0.3 for Zn, 0.003 for Cr, 0.001 for Cd and 0.0003 for As [56]. No significant risk and non-carcinogenic effects are expected if the value is smaller than one (*HI* < 1). However, if the *HI* value is higher than one (*HI* > 1), non-carcinogenic risk effects may arise [30]. 

According to USEPA [57], Cd, Cr, Pb and As are classified into heavy metals that induce carcinogenic risk. Therefore, the carcinogenic risk or the lifetime cancer risk (*LCR*) is calculated as follow:

(14) Cancer risk=CDI×CSF

(15) $$\begin{array}{c} \sum Cancer\;risk=LCR\\ \;\;\;\;\;\;=Cancer\;risk_{ing}+Cancer\;risk_{inh}+Cancer\;risk_{dermal} \end{array}$$

where *LCR* is the summation of the cancer risk from each exposure pathway. The values of cancer slope factor (*CSF*) for Cd, Cr, Pb and As are 6.3, 0.5, 0.0085 and 1.5 mg/kg/day, respectively [56]. The tolerable threshold value of the cancer risk is 1 × 10^−4^, while the acceptable *LCR* for regulatory purposes is 1 × 10^−6^ to 1 × 10^−4^ [55].

### 2.5. Statistical Analyses

Pearson simple-linear regression analysis is conducted to investigate the correlation among these metals. The principal component analysis (PCA) is used to identify the potential sources. 

(1) Pearson correlation coefficient

The Pearson correlation coefficient of two variables is calculated below:

(16) $$\rho =\frac{Cov(X,Y)}{\sigma_{X}\sigma_{Y}}$$

where *ρ* is the correlation coefficient; *Cov* (*X, Y*) is the covariance of the two variables; *σ_X_* and *σ_Y_* is the standard deviation of the variable *X* and *Y*, respectively.

The transformed *ρ* follows t distribution with *n*–2 degrees of freedom [58] as described as follows.

(17) $$T=\frac{\rho \sqrt{n-2}}{\sqrt{1-\rho^{2}}}$$

(2) Principal component analysis 

The principal component analysis, PCA, is a specific linear transformation based on multi original variables [59]. A vector with dimension P is described in the following.

(18) $$X=(X_{1},X_{2},X_{3},\ldots X_{p}{)}^{\prime }$$

The linear transformation is described below.

(19) $$\begin{array}{l} Z_{1}={a_{1}}^{\prime }X=a_{11}X_{1}+a_{21}X_{2}+a_{31}X_{3}+\ldots +a_{p1}X_{p}\\ Z_{2}={a_{2}}^{\prime }X=a_{12}X_{1}+a_{22}X_{2}+a_{32}X_{3}+\ldots +a_{p2}X_{p}\\ .....................................................................\\ Z_{p}={a_{p}}^{\prime }X=a_{1p}X_{1}+a_{2p}X_{2}+a_{3p}X_{3}+\ldots +a_{pp}X_{p} \end{array}$$

In order to maintain the transformed variable, *Z*, as representing the maximum information, the *Z_i_* is independent from one another.

(20) $$Cov(Z_{i},Z_{j})=0$$

(3) The rank sum test

If the two populations with the number of *n*_1_ and *n*_2_ have the same continuous distribution, then mean of the rank sum follows the normal distribution. Thus, the *z* stat is obtained by a normalized transformation [59] described in the following:

(21) $$z=\frac{\mu_{W}-\sigma_{w}}{\sigma_{w}}=\frac{\frac{n_{1}(N\;+\;1)}{2}-\sqrt{\frac{n_{1}n_{2}(N\;+\;1)}{12}}}{\sqrt{\frac{n_{1}n_{2}(N\;+\;1)}{12}}}$$

where *μ_w_* and *σ_w_* are mean and standard deviation of the rank sum, *N* = *n*_1_ + *n*_2._ The significant level is obtained based on the *z* stat, e.g., α = 0.05 with *z* stat of 1.96. 

## 3. Results 

### 3.1. Spatial and Temporal Trends of Heavy Metals

#### 3.1.1. Temporal Changes 

**Figure 2:**
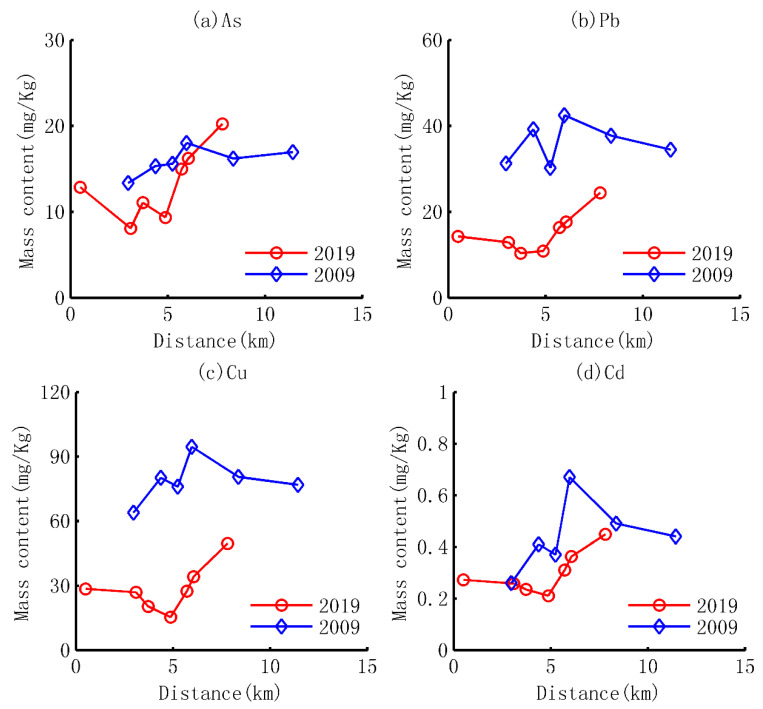

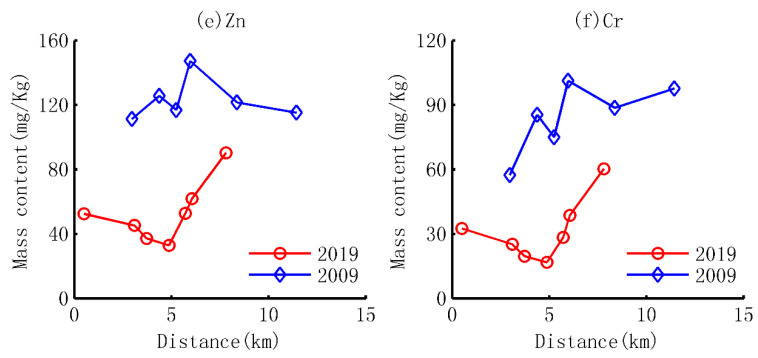
Comparisons on the average content of heavy metals between 2019 measured in this study and 2009 from literature [32] following the water flow direction; (**a**) As; (**b**) Pb; (**c**) Zn; (**d**) Cd; (**e**) Zn; (**f**) Cr.

Based on the current values measured in this study and the values reported in 2009, all the heavy metals in sediment decreased dramatically in Huaxi Reservoir after a ten-year development, as shown in Table 1 and Figure 2. 

In 2019, the average total concentrations of As, Pb, Cu, Cd, Zn and Cr are 14.10 mg/kg, 13.26 mg/kg, 29.32 mg/kg, 0.29 mg/kg, 55.58 mg/kg and 31.33 mg/kg, respectively. The average concentration of heavy metals in sediment of Huaxi Reservoir feature the following order: Cd < As < Pb < Cu < Cr < Zn. Meanwhile, all the contents of these heavy metals are lower than their background values in the study area (Guizhou Province), as is shown in Table 1. 

Compared with the heavy metal contents in 2009, the average content of heavy metals in sediment has decreased by 16%, 59%, 63%, 34%, 54% and 62% which corresponds to As, Pb, Cu, Cd, Zn and Cr, respectively. In 2009, the average contents of Pb, Cu, Zn in the sediments were higher than their background values, especially Cu (78.63 vs. 35.2 mg/kg) and Zn (122.94 vs. 99.5 mg/kg), while the other heavy metals such as As, Cd, Cr were lower than their background contents. The higher value than the background value suggests that Pb, Cu, Zn are mainly impacted by anthropogenic activities before the tools were taken [60,61]. In 2019, however, the average contents of all the six heavy metals have decreased and are lower than their background value, which shows that the external source of specific heavy metals such as Pb, Cu and Zn has been cut off in the upstream areas of the reservoir after the ten-year development.

The differences of these heavy metals in content between 2019 and that of 2009 are identified by a widely used nonparametric test and rank sum test with significant levels of α = 0.05, as shown in Table 2. The results show that all of the heavy metals display a significant decreasing trend except As, with the significant level of 0.09. Despite this result, the relative decrement of 16% reflects an obvious decrease trend of As. 

#### 3.1.2. Spatial Distributions and Their Affecting Factors

Based on the average content of heavy metals in the sediment sampled from seven profiles following the water flow direction, the spatial distribution patterns of heavy metals are also shown in Figure 2 and the stats are given in Table 2. 

##### Spatial Distributions

The spatial distributions of all six heavy metals in sediment, generally, display the trend of decrease first and then increase from the upstream to downstream in 2019. The decrease trend begins from the first sampling site, S1, and ends at the second sites, S2, for As and at the fourths sampling site, S4, for the other heavy metals. After that, the average contents of heavy metals rise with the water flow direction. However, the different spatial patterns are observed in 2009 and are also shown in Figure 2. The contents of heavy metals show the trend of increase first and then gradual decrease. The maximum values of all six heavy metals are observed at Zhengshan Village, sampling site 6 (S6) in Figure 1. By the comparisons between 2019 and 2009, it can be concluded that the spatial distributions changes substantially after the ten-year development in the upstream areas of the dam.

##### Affecting Factors

The decrease pattern is usually affected by the input of pollution sources. Conversely, the increase trend shows a similar pattern to the natural river system that is not contaminated and the increase in heavy metals is mainly affected by the finer texture of sediment from the upstream to downstream.

**Figure 3:**
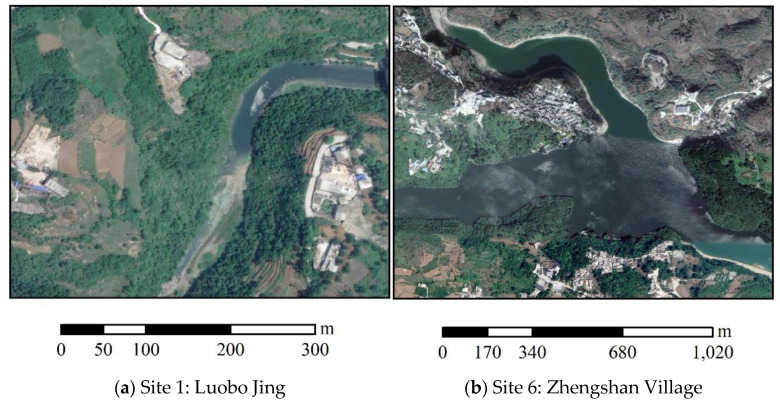
Remote sensing images from Google Earth around high value sampling sites in 2019.

Based on the investigation, the high value in 2009 can be mainly attributed to the non-point source pollution, such as domestic sewage; agricultural pollution such as pesticide and fertilizer; and soil erosion from infrastructure construction. Moreover, Zhengshan Village is a tourist attraction which results in pollution more or less before the industrial restructuring. In the downstream of Zhengshan Village, the content of heavy metals displays a decreasing trend following water flow direction, which is different from the patterns in 2019. Furthermore, the high value at the first sampling site is mainly a result from agricultural production and infrastructure construction, as shown in Figure 3a. The heavy metals of background soil from the agricultural production and construction sites enter the reservoir with surface runoff in rainy days and then results in the high values in the sediment. In fact, the average contents of these heavy metals in the sediment are less than their background values in Guizhou Province (as shown in Table 1), although the contents of the first sample are higher than the following several samples.

### 3.2. Assessment of Heavy Metal Contamination

#### 3.2.1. Sediment Quality Guidelines

Based on the calculated *TEL*, it can be observed that the current sediment toxicity of heavy metals is much smaller than that of ten years ago, as shown in Table 1. In 2009, the average concentration of As, Pb, Cu and Cr in sediment exceeded the *TEL* value while was less than the *PEL* value that could have adverse effect on sediment-dwelling organisms. After the ten-year industrial restructuring, all of the four exceeded heavy metals have decreased to less than *TEL* value with the exception of As. Normally, As is considered as the most dangerous trace metal in terms of environmental concern due to its high potential toxicity [22,62]. As shown in Table 1, the contents of As in sediment exceeds the *TEL* value in 2019, implying some threat to the sediment-dwelling organisms. 

The high concentration of As is mainly associated with various anthropogenic activities such as chemical fertilizers, arsenical pesticides, domestic and industrial wastes. In 2009, the high background and anthropogenic activities could be the main reasons. The average Pb concentration is high in sediments which might be due to the vehicle emissions, domestic wastes and agricultural chemicals. The average concentration of Cu was higher in sediments due to the fragmentation of the rocks in the river basins, the activities of the stone pebbles, intensive utilization of pesticides and fertilizers for crop production and the urban wastes disposal [2,22,63]. Inorganic fertilizers, atmospheric deposition and sewage sludge were found to be the main sources of Cr [64]. 

#### 3.2.2. Geo-Accumulation Index (*I_geo_*) of Metals

**Figure 4:**
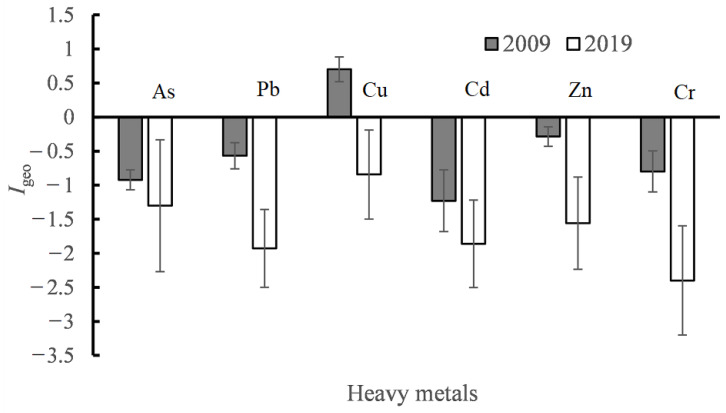
*I_geo_* of heavy metals calculated from the data in 2019 and that of 2009 from literature [32].

The results of *I*_geo_ are shown in Figure 4. It can be observed that the average *I*_geo_ also decreases dramatically after ten years of environmental protection and the average *I*_geo_ has decreased to an unpolluted level with a value of less than zero in 2019. In particular, the *I*_geo_ of Cu has decreased from a positive to negative value. 

In addition, the ranking of *I_geo_* for the six heavy metals has changed. It follows the order of Cr < Pb < Cd < Zn < As < Cu in 2019 while it followed the order Cd < As < Cr < Pb < Zn < Cu in 2009. The only change in order of *I_geo_* is As, from the fifth to second. The highest *I_geo_* value of Cu is mainly from the infrastructure constructions and their ancillary industries, such as quarrying and grease. 

#### 3.2.3. Contamination Factor (*CF*) and Potential Ecological Risk Index (*E^i^_r_*)

**Figure 5:**
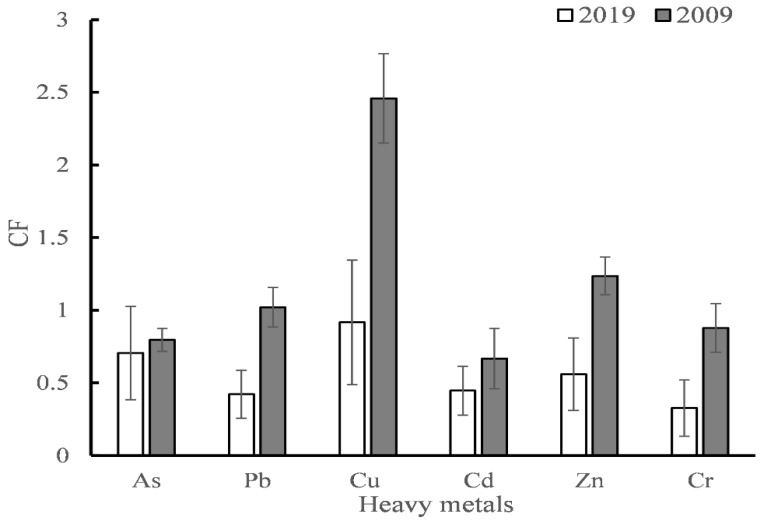
CF values of heavy metals calculated from data in 2019 and that of 2009 from literature [32].

The contamination factor (*CF*) values of all metals in 2019 and 2009 are presented in Figure 5. It can be found that each *CF* is less than 1 in 2019. However, the average values of Pb, Cu and Zn were larger than 1 in 2009. The decreasing trend shows that some heavy metals such as Pb, Cu and Zn have decreased from the moderate contamination degree (1 < *CF* < 3) to low degree of contamination (*CF* < 1) after ten years. 

**Figure 6:**
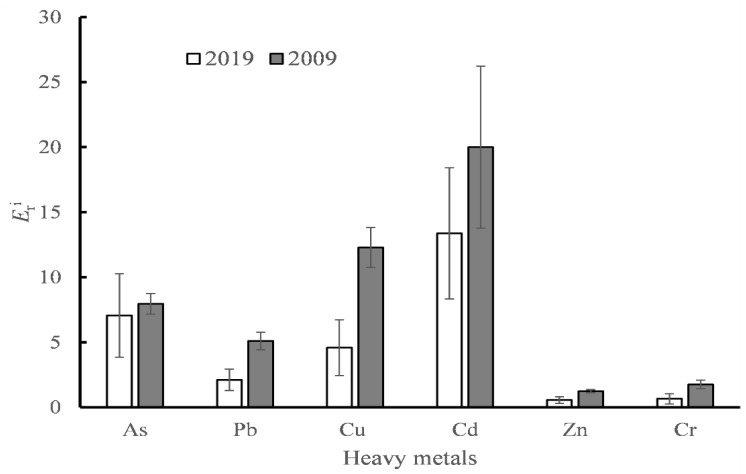
$E_{r}^{i}$ value of heavy metals calculated from data in 2019 and that of 2009 from literature [32].

Graphical representations of the $E_{r}^{i}$ values are shown in Figure 6. Accordingly, all heavy metals in sediments display low levels of potential ecological risk ($E_{r}^{i}$ < 40) in both 2019 and 2009. Moreover, the $E_{r}^{i}$ values of all metals in sediments in 2019 have decreased substantially compared to the data from 2009. 

By using the above analysis, the contamination factor and potential ecological risk index have decreased after the ten year sustainable development. However, the average concentration of As is still larger than *TEL*, implying some threat to the sediment-dwelling organisms health. 

#### 3.2.4. Potential Acute Toxicity of Metals

**Figure 7:**
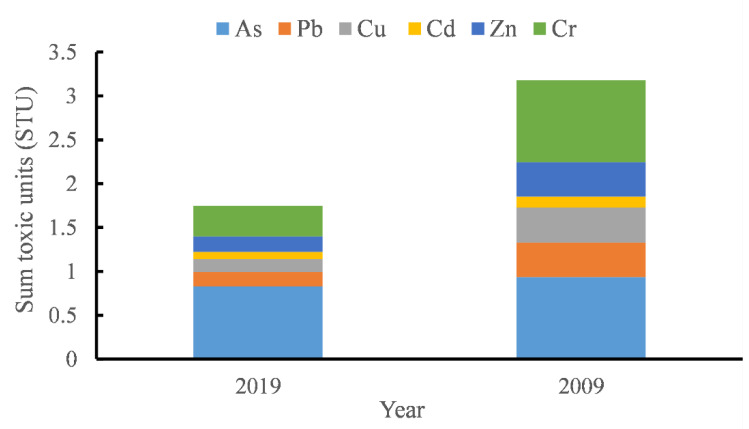
Sum of the toxic units in surface sediments of Huaxi Reservoir based on the data in 2019 and that of 2009 from literature [32].

Potential acute toxicity of metals in sediments can be estimated as the sum of the toxic units (STU) calculated as the ratio of the determined concentration of toxic metals to the probable effect levels (*PEL*s) value [65]. The levels of toxicity are divided into three classes: low toxicity level with STU less than 4; moderate toxicity level with STU between 4 and 6; and heavy toxicity level with STU larger than 6 [66]. The sum of toxic units for toxic metals in the sediments of Huaxi Reservoir is presented in Figure 7. It can be found that both the sums of toxic units at Huaxi Reservoir in 2009 and 2019 are not greater than 4 which indicates low toxicity level of heavy metals to sediment-dwelling fauna in this area [67,68].

#### 3.2.5. *EF*, *mHQ*, *ECI* and *TRI* Values 

The values of *EF*, *mHQ*, *ECI* and *TRI* are presented in Table 3. The average *EF* is 1.32, 1.02, 0.81, 0.64, 0.61 and 0.47 in 2019 corresponding to the heavy metal of Cu, As, Zn, Cd, Pb and Cr, respectively. Generally, the value of *EF* less than 1 refers to the content of a given heavy metal entirely coming from earth crust or natural weathering processes, while an *EF* value greater than 1.5 indicates that the considerable volume of the heavy metal more likely results from anthropogenic processes [22]. In this study, the *EF*s of all the heavy metal are less than 1.5 and the *EF*s of the four metals (Zn, Cd, Pb and Cr) are less than 1. The *EF* values suggests that a natural source is the main cause of the enrichment of all metals in sediments of Huaxi Reservoir. The *EF*s of 2009 were not calculated due to the lacking concentration of *Fe*.

In addition, both *ECI* and *TRI* show the substantial decreasing trend. The former, *ECI*, from 1.78 to 1.29, implies the uncontaminated level (*ECI* < 2) in both 2019 and 2009. The latter, *TRI* from 7.39 to 4.03, implies the risk shifting from the low toxic risk in 2009 to no toxic risk in 2019.

The calculated *mHQ* exhibits some differences. In 2019, no heavy metal shows considerable severity of contamination, one metalloid (As) shows the moderate severity of contamination, two heavy metals (Cr and Cu) show low severity of contamination and three heavy metals (Pb, Cd and Zn) show very low severity of contamination. In 2009, Cd shows very low severity, As shows considerable severity and the other four heavy metals show moderate severity. Thus, the *mHQ* shows that contamination still cannot be neglected, especially the risk posed from As.

#### 3.2.6. Health Risk Evaluation of Metals

The *CDI*, *HQ* and *HI* for non-carcinogenic risk of metals from the three exposure pathways on adults and children are presented in Table 4.

As for non-carcinogenic risk, it mainly shows the following two characteristics. The first that all of the *HI* values are lower than one, indicating no significant risk of non-carcinogenic effects of the heavy metals in both 2019 and 2009 and the much smaller values in 2019 shows the positive effect of the development in the past ten years. Secondly, *HI* values of both children and adult follow the order of As > Cr > Pb > Cu >Cd > Zn in 2009 and 2019.

The *LCR* value of Pb, Cd, Cr and As in sediments of Huaxi Reservoir are presented in Table 5. The results show that the *LCR* of all four heavy metals are lower than the threshold value of 1 × 10^−4^ in 2019 for both adults and children. Although the *LCR* of Cr for children is 1.35 × 10^−4^ in 2009, it has decreased to 5.02 × 10^−5^ in 2019. Thus, it can be concluded that there is no carcinogenic risk from the heavy metals in sediments after the ten-year development.

### 3.3. Source Analysis of Heavy Metals

In order to further identify the potential sources of heavy metals in the sediment of Huaxi Reservoir, the Pearson’s correlation coefficient (PCC) analysis and principal component analysis (PCA) are used to uncover more details.

As shown in Table 6, most of the heavy metal pairs exhibit a significant positive correlation at the significant level of 0.05, with the minimum correlation coefficient of 0.52 in 2009 and 0.65 in 2019. The high correlation coefficient indicates that two metals are possibly originated from the same resources. A larger value in 2019 implies the main source of pollution accounts for a higher proportion than before.

The results of PCA are given in Table 7. It can be found that only one main factor is identified and the first principal component accounts for 86.07% and 86.83% of the variance in the dataset of 2019 and 2009, respectively. The results indicate that the heavy metals in the sediment of the Huaxi Reservoir are derived from similar sources. A similar conclusion has also been obtained from a higher correlation between the variables in 2019.

The combined results of the Pearson’s correlation coefficient analysis, principal component analysis and background data suggest that heavy metal concentrations are mainly from natural sources in the Huaxi Reservoir.

## 4. Discussions

### 4.1. Different Spatial Patterns Following Water Flow Direction

(1) Lower vs. higher than background value

In this study, it was found that the content of heavy metals in Huaxi Reservoir is lower than their background value. However, the contents of most metals in the sediment of the other three large reservoirs (Hongfeng Lake, Baihua Lake and Aha Lake) in the city (Guiyang) are higher than their background value (Table 1). The higher values are mainly the result of human activities [36,37,38,69]. Compared with the other three, reducing the internal pollution of heavy metals in Huaxi Reservoir has, thus. achieved great success after the ten-year sustainable development.

(2) Decrease-increase vs. increase decrease pattern

The spatial patterns of heavy metals in a natural river, generally, display an increasing trend following the water flow direction. However, the decreasing trend from the pollution source to the downstream area is widely observed in the heavy contaminated river, lake or reservoir. For example, Hongfeng Lake located about 40 km east of the study area reflects the decreasing trend [69]. In this study, the spatial distribution of these heavy metals generally exhibits a decrease-increase pattern following water flow in 2019 while an opposite pattern is observed in 2009. The different patterns imply that the input of the main pollution source has shifted from the middle to upstream areas.

### 4.2. Differences among These Risk or Pollution Indices

The pollution or risk indices calculated from data measured in 2019 are given in Table 8. Nine indices exhibit the lowest level and possess good quality relative to the water body. The two exceptions are *TEL* and *mHQ*. The content of As is larger than *TEL* while less than *PEL*, implying some threat to the sediment-dwelling organisms. The *mHQ* shows the level of very low to moderate severity contaminations and As is the highest severity of these heavy metals.

The different level among these indices, generally, is the result of their formulas and is manifested by the following aspects.

(1) If these indices are calculated from the values of the background or the reference metal, then the results usually show the lowest level, e.g., *I_geo_*, *CF*, *E*_r_^i^ and *EF*. In this study, the background values of the heavy metals are higher than the levels in many other areas in China and the rest of the world.

(2) If the indices are calculated from the total or average value of these elements, the results also show the lowest level, e.g., *TRI*, *ECI*, *HQ* and *LCR*. The reason is the complement from different elements. The negative effect of some element such as As may by complemented by the positive effect of some low content element such as Cd.

(3) If the indices are calculated from the content of each element and based on the absolute value instead of removing the effect of the background value, the risk still exists more or less, e.g., *mHQ* and *TEL*. Here, the high background value of As may exert some risk to the water environment.

After the ten-year development, the risk of heavy metals in the sediment of Huaxi Reservoir cannot be neglected, although many indices show the lowest level.

### 4.3. Source Analysis and Management in the Future

(1) Source analysis

The source analysis above has revealed only one main source of heavy metals in the sediment in both 2019 and 2009. In other words, the natural source dominates the content of heavy metals in sediment. However, the spatial pattern of the increasing trend first and then the decreasing trend in 2009 indicates an obvious external source input in Zhengshan Village located in the middle of the reservoir. At that time, the main external source was agriculture, infrastructure construction and the outdoor barbecuing. However, both the PCC and PCA failed to identify the external source.

Here, the reason for the failure of the two methods can be attributed to the shortage of their algorithms. Both the PCA and PCC, generally, are based on the average or total trend. Similar to the indices such as *TRI*, *ECI*, *HQ* and *LCR* above, the negative effect from one element may by complemented by another positive effect.

(2) Management in the future

The ten-year sustainable development has substantially reduced the heavy metals in the sediment of the Huaxi Reservoir. In 2009, the contents of Pb, Cu and Cr in the Huaxi Reservoir are close to or larger than those in the other reservoirs around the study area [36,37,38]. In 2019, the contents of these elements are much lower in study area. Thus, the success of the Huaxi Reservoir can provide references to protect the other reservoirs in the Karst area of southwestern China.

However, the challenge to water environment protection still cannot be neglected in the future. The study area is located in a geochemically sensitive and ecologically fragile karst carbonate rock region with a higher background value of heavy metals. Human activities such as agricultural production and infrastructure construction would promote the weathering of the rocks and soil. The heavy metals are adsorbed by the soil particles and then been transported into the river accompanied with the flow of surface or underground runoff driven by rainfall. These non-point source inputs of heavy metals from the high background value soil cannot be easily controlled by promulgating a series of bans.

## 5. Conclusions

By comparing the contents, spatial distributions, risk indices and sources of metals in 2019 with that of the information from 2009, the conclusion are as follows.

(1) The contents of heavy metals in sediment have decreased dramatically in Huaxi Reservoir and the average reduction rate is 16%, 59%, 63%, 34%, 54% and 62% corresponding to As, Pb, Cu, Cd, Zn and Cr, respectively. The spatial distribution of these metals generally exhibits a decrease-increase pattern following water flow in 2019, while an opposite pattern was observed in 2009. The different patterns imply that the input of the main pollution source has shifted from the middle to upstream areas.

(2) The water environment risk indices have decreased substantially. The indices based on the total content, average content and background value have decreased to the lowest level. However, those indices calculated from the content of each element, e.g., *mHQ* and *TEL*, indicate that the risk still cannot be neglected. The difference is mainly from the high background value of As and Cr, which are higher than *PEL*.

Therefore, the ten-year sustainable development has achieved significant progress in water environmental protection. However, the high background value of specific heavy metals, e.g., As and Cr, would still exert some risk to the water environment protections and the non-point source input of heavy metal from the weathering cannot be controlled easily by promulgating a series of bans. These results will provide important reference for creating the policies of water environment protection, especially in the Karst area of southwestern China where heavy metals exhibit high background values.

## Figures and Tables

**Table 1:** Concentrations of heavy metals in the sediments of the Huaxi Reservoir and other nearby reservoirs, the background values and sediment quality guidelines (mg/kg).

Reservoir (Lake) and Site	Stats	As	Pb	Cu	Cd	Zn	Cr	Reference
Huaxi Reservoir, Guiyang, China (2019)	Mean	13.26	14.84	29.32	0.29	55.58	31.33	This study
	SD	6.00	5.43	12.82	0.10	23.22	17.43	
	Min	2.75	7.16	15.02	0.11	24.19	12.07	
	Max	22.34	26.12	35.91	0.48	95.12	70.89	
Huaxi Reservoir, Guiyang, China (2009)	Mean	15.91	35.91	78.63	0.44	122.94	84.19	[32]
	SD	1.45	4.35	8.99	0.12	11.80	14.69	
	Min	13.35	31.25	63.91	0.26	111.21	57.38	
	Max	18.01	42.50	94.51	0.67	147.24	101.21	
Aha Reservoir, Guiyang, China	Mean	26.28	75.87	59.53	1.12	164.65	104.86	[36]
Baihua Lake, Guiyang, China	Mean	-	40.00	68.00	0.95	339.00	66.00	[37]
Hongfeng Lake, Guiyang, China	Mean	29.70	35.90	9.19	0.77	142.00	87.90	[38]
Element value of sediments in China	Mean	9.1	25	21	0.14	68	38	[39]
Guizhou soil background	Mean	20.00	35.20	32.00	0.66	99.50	95.90	[40]
*TEL*	Mean	5.9	35	35.7	0.6	123	37.3	[35]
*PEL*	Mean	17	91.3	197	3.53	315	90	[35]
*SEL*	Mean	33	250	110	10	820	110	[35]

**Table 2:** The results from the rank sum test with significant level of *α* = 0.05 between the average content of heavy metals in 2019 and that of 2009 from literature [32].

Heavy Metals	As	Pb	Cu	Cd	Zn	Cr
*p* value	0.0903	0.0006	0.0006	0.0256	0.0006	0.0012
H	0	1	1	1	1	1
*Z* stat	−1.69	−3.44	−3.44	−2.23	−3.44	−3.25
Rank sum	39	28	28	35	28	29

**Table 3:** *mHQ*, *ECI* and *TRI* values for heavy metals in stream sediments of Huaxi Reservoir.

	Index	As	Pb	Cu	Cd	Zn	Cr
2009	*mHQ*	2.03 ± 0.10	1.25 ± 0.08	1.82 ± 0.11	0.95 ± 0.15	1.24 ± 0.06	1.99 ± 0.19
	*ECI*	1.78 ± 0.09					
	*TRI*	7.39 ± 1.01					
2019	*EF*	1.02 ± 0.46	0.61 ± 0.24	1.32 ± 0.62	0.64 ± 0.24	0.81 ± 0.36	0.47 ± 0.28
	*mHQ*	1.91 ± 0.50	0.80 ± 0.15	1.11 ± 0.24	0.78 ± 0.16	0.83 ± 0.17	1.21 ± 0.35
	*ECI*	1.29 ± 0.08					
	*TRI*	4.03 ± 1.70					

Notes: the results of 2009 are calculated form the data of literature [32].

**Table 4:** Chronic daily intake (*CDI*, mg/kg/day), hazard quotient (*HQ*) and cumulative hazard index (*HI*) for non-carcinogenic risk.

People	Heavy Metals				2009							2019			
		*CDI_ing_*	*CDI_inh_*	*CDI_derma_*	*HQ_ing_*	*HQ_inh_*	*HQ_dermal_*	*HI*	*CDI_ing_*	*CDI_inh_*	*CDI_derma_*	*HQ_ing_*	*HQ_inh_*	*HQ_dermal_*	*HI*
Adult	As	2.2 × 10^−^^5^	3.2 × 10^−^^9^	5.2 × 10^−^^9^	7.3 × 10^−^^2^	1.1 × 10^−^^5^	1.7 × 10^−^^5^	7.3 × 10^−^^2^	1.9 × 10^−^^5^	2.8 × 10^−^^9^	4.6 × 10^−^^9^	6.4 × 10^−^^2^	9.5 × 10^−^^6^	1.6 × 10^−^^5^	6.4 × 10^−^^2^
	Pb	4.9 × 10^−^^5^	7.2 × 10^−^^9^	1.2 × 10^−^^8^	1.4 × 10^−^^2^	2.1 × 10^−^^6^	3.4 × 10^−^^6^	1.4 × 10^−^^2^	2.3 × 10^−^^5^	3.0 × 10^−^^9^	4.9 × 10^−^^9^	5.8 × 10^−^^3^	8.5 × 10^−^^7^	1.4 × 10^−^^6^	5.8 × 10^−^^3^
	Cu	1.1 × 10^−^^4^	1.6 × 10^−^^8^	2.6 × 10^−^^8^	2.0 × 10^−^^3^	4.3 × 10^−^^7^	7.0 × 10^−^^7^	2.9 × 10^−^^3^	4.2 × 10^−^^5^	5.9 × 10^−^^9^	9.6 × 10^−^^9^	1.9 × 10^−^^3^	1.6 × 10^−^^7^	2.6 × 10^−^^7^	1.9 × 10^−^^3^
	Cd	6.0 × 10^−^^7^	8.9 × 10^−^^11^	1.5 × 10^−^^10^	6.0 × 10^−^^4^	8.9 × 10^−^^8^	1.5 × 10^−^^7^	6.0 × 10^−^^4^	4.0 × 10^−^^7^	5.9 × 10^−^^11^	9.7 × 10^−^^11^	4.0 × 10^−^^4^	5.9 × 10^−^^8^	9.7 × 10^−^^8^	4.0 × 10^−^^4^
	Zn	1.7 × 10^−^^4^	2.5 × 10^−^^8^	4.0 × 10^−^^8^	5.6 × 10^−^^4^	8.3 × 10^−^^8^	1.4 × 10^−^^7^	5.6 × 10^−^^4^	7.6 × 10^−^^5^	1.1 × 10^−^^8^	1.8 × 10^−^^8^	2.5 × 10^−^^4^	3.7 × 10^−^^8^	6.1 × 10^−^^8^	2.5 × 10^−^^4^
	Cr	1.2 × 10^−^^4^	1.7 × 10^−^^8^	2.8 × 10^−^^8^	3.8 × 10^−^^2^	5.7 × 10^−^^6^	9.2 × 10^−^^6^	3.9 × 10^−^^2^	4.3 × 10^−^^5^	6.3 × 10^−^^9^	1.0 × 10^−^^8^	1.4 × 10^−^^2^	2.1 × 10^−^^6^	3.4 × 10^−^^6^	1.4 × 10^−^^2^
Children	As	5.1 × 10^−^^5^	9.0 × 10^−^^8^	6.1 × 10^−^^9^	1.7 × 10^−^^1^	3.0 × 10^−^^4^	1.7 × 10^−^^5^	1.7 × 10^−^^1^	4.5 × 10^−^^5^	8.0 × 10^−^^8^	5.4 × 10^−^^9^	1.5 × 10^−^^1^	2.7 × 10^−^^4^	1.6 × 10^−^^5^	1.5 × 10^−^^1^
	Pb	1.2 × 10^−^^4^	2.0 × 10^−^^7^	1.4 × 10^−^^8^	3.3 × 10^−^^2^	5.8 × 10^−^^5^	3.4 × 10^−^^6^	3.3 × 10^−^^2^	4.7 × 10^−^^5^	8.4 × 10^−^^8^	5.7 × 10^−^^9^	1.4 × 10^−^^2^	2.4 × 10^−^^5^	1.4 × 10^−^^6^	1.4 × 10^−^^2^
	Cu	2.5 × 10^−^^4^	4.4 × 10^−^^7^	3.0 × 10^−^^8^	6.8 × 10^−^^3^	1.2 × 10^−^^5^	7.0 × 10^−^^7^	6.8 × 10^−^^3^	9.4 × 10^−^^5^	1.7 × 10^−^^7^	1.1 × 10^−^^8^	2.5 × 10^−^^3^	4.5 × 10^−^^6^	2.6 × 10^−^^7^	2.5 × 10^−^^3^
	Cd	1.4 × 10^−^^6^	2.5 × 10^−^^9^	1.7 × 10^−^^10^	1.4 × 10^−^^3^	2.5 × 10^−^^6^	1.5 × 10^−^^7^	1.4 × 10^−^^3^	9.4 × 10^−^^7^	1.7 × 10^−^^9^	1.1 × 10^−^^10^	9.4 × 10^−^^4^	1.7 × 10^−^^6^	9.7 × 10^−^^8^	9.4 × 10^−^^4^
	Zn	3.9 × 10^−^^4^	6.9 × 10^−^^7^	4.7 × 10^−^^8^	1.3 × 10^−^^3^	2.3 × 10^−^^6^	1.4 × 10^−^^7^	1.3 × 10^−^^3^	1.8 × 10^−^^4^	3.1 × 10^−^^7^	2.1 × 10^−^^8^	5.9 × 10^−^^4^	1.1 × 10^−^^6^	6.1 × 10^−^^8^	5.9 × 10^−^^4^
	Cr	2.7 × 10^−^^4^	4.8 × 10^−^^7^	3.2 × 10^−^^8^	9.0 × 10^−^^2^	1.6 × 10^−^^4^	9.2 × 10^−^^6^	9.0 × 10^−^^2^	1.0 × 10^−^^4^	1.8 × 10^−^^7^	1.2 × 10^−^^8^	3.3 × 10^−^^2^	5.9 × 10^−^^5^	3.4 × 10^−^^6^	3.3 × 10^−^^2^

Note: the results of 2009 are calculated form the data of literature [32].

**Table 5:** Carcinogenic risk of different exposure pathways for adult and children.

Year	Heavy Metals	Adult				Children			
		CR_*Ing*	CR_*Inh*	CRD	*LCR*	CR_*Ing*	CR_*Inh*	CRD	*LCR*
2009	Cd	3.8 × 10^−6^	5.6 × 10^−10^	9.1 × 10^−10^	3.8 × 10^−6^	8.9 × 10^−6^	1.6 × 10^−8^	1.1 × 10^−9^	8.9 × 10^−6^
	Cr	5.8 × 10^−5^	8.5 × 10^−9^	1.4 × 10^−8^	5.8 × 10^−5^	1.4 × 10^−4^	2.4 × 10^−7^	1.6 × 10^−8^	1.4 × 10^−4^
	Pb	4.2 × 10^−7^	6.2 × 10^−11^	1.0 × 10^−10^	4.2 × 10^−7^	9.8 × 10^−7^	1.7 × 10^−9^	1.2 × 10^−10^	9.8 × 10^−7^
	As	3.3 × 10^−5^	4.8 × 10^−9^	7.8 × 10^−9^	3.3 × 10^−5^	7.6 × 10^−5^	1.4 × 10^−7^	9.2 × 10^−9^	7.6 × 10^−5^
2019	Cd	2.5 × 10^−6^	3.7 × 10^−10^	6.1 × 10^−10^	2.5 × 10^−6^	5.9 × 10^−6^	1.6 × 10^−8^	7.1 × 10^−10^	5.9 × 10^−6^
	Cr	2.2 × 10^−5^	3.2 × 10^−9^	5.2 × 10^−9^	2.2 × 10^−5^	5.0 × 10^−5^	8.8 × 10^−8^	6.0 × 10^−9^	5.0 × 10^−5^
	Pb	1.7 × 10^−7^	2.5 × 10^−11^	4.2 × 10^−11^	1.7 × 10^−7^	4.0 × 10^−7^	7.1 × 10^−10^	4.8 × 10^−11^	4.0 × 10^−7^
	As	2.9 × 10^−5^	4.3 × 10^−9^	7.0 × 10^−9^	2.9 × 10^−5^	6.8 × 10^−5^	1.2 × 10^−7^	8.1 × 10^−9^	6.8 × 10^−5^

Notes: CR_*Ing*: *Cancer risk _ingestion_*; CR_*Inh*: *Cancer risk _inhalation_*;. CRD: *Cancer risk _dermal_*; *LCR*: Lifetime cancer risk; the results of 2009 are calculated form the data of literature [32].

**Table 6:** Pearson’s correlation matrix for the heavy metal concentrations in the surface sediments of the Huaxi Reservoir in 2019 and 2009.

Year	Heavy Metals	As	Pb	Cu	Cd	Zn	Cr
2019	As	1.00					
	Pb	0.70	1.00				
	Cu	0.65	0.96 **	1.00			
	Cd	0.69	0.91 **	0.93 **	1.00		
	Zn	0.52	0.84 **	0.91 **	0.96 **	1.00	
	Cr	0.69	0.98 **	0.98 **	0.86 **	0.81 *	1.00
2009	As	1.00					
	Pb	0.65	1.00				
	Cu	0.89 *	0.85 *	1.00			
	Cd	0.92 **	0.84 *	0.97 **	1.00		
	Zn	0.71	0.87 *	0.93 **	0.90 *	1.00	
	Cr	0.95 **	0.74	0.85 *	0.87 *	0.65	1.00

Note: * means significant level of 0.05; ** means significant level of 0.01; the results of 2009 are calculated form the data of literature [32].

**Table 7:** Result of principal components matrix for metals of Huaxi Reservoir in 2019 and 2009.

Heavy Metals	2019	2009
	Factor 1	Factor 1
As	0.75	0.92
Pb	0.97	0.88
Cu	0.98	0.98
Cd	0.97	0.99
Zn	0.91	0.91
Cr	0.96	0.91
Eigenvalue	5.16	5.21
% of variance	86.07	86.83

Note: the results of 2009 are calculated form the data of literature [32].

**Table 8:** Summary for different pollution or risk indices of heavy metals in sediment of Huaxi Reservoir in 2019.

Indices	Risk or Pollution Level	Description
*I_geo_*	Lowest level	Unpolluted
*TEL*	Between *TEL* and *PEL*	**As: some threat to the sediment-dwelling organisms**
*I_geo_*	Lowest level	Unpolluted
*CF*	Lowest level	low degree of contamination
*PEL*	Lowest level	STU < 4, low toxicity level
*EF*	Lowest level	no enrichment
*mHQ*	2nd–4th lowest	Very low to moderate severity of contamination **As: moderate severity** **Cr, Cu: low severity** Pb, Zn, Hg: very low severity
*ECI*	Lowest level	Uncontaminated
*TRI*	Lowest level	No toxic risk
*HI*	Less than threshold value of 1	No significant risk of non-carcinogenic risk effects
*LCR*	Less than threshold value of 1 × 10^−4^	Acceptable *LCR*
